# Influence of Laser Beam Power on Microstructure and Microhardness of Fe/ZrC Coatings Produced on Steel Using Laser Processing—Preliminary Study on the Single Laser Tracks

**DOI:** 10.3390/ma15030758

**Published:** 2022-01-19

**Authors:** Dariusz Bartkowski

**Affiliations:** Institute of Materials Technology, Faculty of Mechanical Engineering, Poznan University of Technology, ul. Piotrowo 3, 61-138 Poznan, Poland; dariusz.bartkowski@put.poznan.pl; Tel.: +48-616-652-665

**Keywords:** laser processing, microstructure, zirconium carbide, coatings, microhardness, chemical composition

## Abstract

This paper presents preliminary tests of the parameter analysis of the Fe/ZrC coatings production process and the obtained properties. The effects of laser beam power on the obtained microstructure, chemical composition and microhardness were investigated. The tests consisted of the production of composite coatings by laser processing of initial coatings made in the form of a paste on a steel substrate. During the tests, a diode laser with a rated power of 3 kW was used. The laser processing process was carried out using a constant scanning speed laser beam of 3 m/min and four different powers of the laser beam: 500 W, 700 W, 900 W, 1100 W. It was found that it is possible to create composite coatings on a steel surface, where the matrix is made of iron-based alloy and the reinforcing phase is ZrC carbide. It was also found that reinforcing phase content decreased as laser beam power increased. A similar relationship was found for microhardness. As laser beam power increases, the microhardness of the iron-based matrix decreases, finally reaching a value lower than the heat-affected zone. It was found that the amount of hard carbide phases in the iron-based matrix affects the total hardness of the coatings. Presented study concern Fe/ZrC coatings that have not previously been produced on steel by laser processing of precoating, which may be a new contribution in the field of metal surface engineering.

## 1. Introduction

Over the last dozen or so years, manufacturing techniques that use a laser beam as a source of energy have developed significantly [1,2,3,4,5]. The laser is used both in the processing of metallic and non-metallic materials. The use of the laser beam for the production of coatings on ferrous (steel, cast iron) [6,7,8,9,10,11,12,13,14] and non-ferrous alloys (mainly nickel and cobalt alloys) [15,16,17,18] is particularly interesting. Recently, laser surface modification techniques have become quite widespread in industrial applications [1,5]. Manufacturers of mining or agricultural tools produce their products in a new, improved form, increasing the durability of tool leading edge with coatings containing hard carbides, usually tungsten carbides. This area is also dealt with by research teams that check the durability of such tools in laboratory and operating conditions [19,20,21]. Coatings reinforced with hard carbide particles usually take the form of a composite. The most frequently used reinforcing phases include tungsten carbide WC and W_2_C [7,8,10,19,20,21,22,23,24,25,26,27,28,29,30], silicon carbide SiC [31,32], boron carbide B_4_C [2,33,34] or titanium carbide TiC [35,36].

Much less attention is given to other types of carbides. Tantalum carbide [12,15,37,38,39,40] and zirconium carbide [41,42,43,44] seem to be very interesting. A mixture of these two carbides forms one of cermets, i.e., ceramic-metal sinters, characterized by very high hardness. There is very little research focusing on zirconium carbide. Literature analysis shows that the idea of producing coatings containing these types of carbides on steel seems to be a novelty. For example, authors working on zirconium carbide focus on gradient structures. In paper [32], the authors created a ZrC-SiC and TC_4_ gradient structure with an additive method using a laser deposition technique and brazing process. The ZrC-SiC coating was produced by hot pressing ZrC and SiC powders at of 2000 °C for 60 min. The authors then investigated the gradient structure by determining the effect of gradient layer thickness on residual stress.

In paper [44], the authors prepared a composite coating on a Cu matrix reinforced with ZrB_2_-ZrC on a copper substrate using laser surfacing and self-propagating high-temperature synthesis reaction (SHS). Zirconium ceramics was synthesized in situ according to the designed SHS reaction within the coatings. The results indicated that the in situ ZrB_2_ ceramics had a needle-like morphology and the ZrB_2_ was covered with dendritic nickel crystals. This nickel formed an intermediate transition layer between the ceramic and the metallic matrix. The submicron particle phases (ZrC) had a rectangular copper matrix morphology and the side length was 500 nm. The composite coating had a dense structure, a high degree of dilution, and excellent metallurgical bonding to the copper substrate. Hard ceramic fibers and particles were synthesized in situ which were uniformly dispersed in the metal matrix, which improved mechanical properties of the coating. The average value of the microhardness of composite coatings was 410 HV0.2 and was almost six times higher than that of copper. The authors also conducted wear tests. The wear mechanism of the composite coating was a combination of abrasive wear and adhesive wear, and the loss of wear volume was approximately 85% less than that of the uncoated substrate. In paper [45], the authors investigated the effect of pure zirconium on strengthening the microstructure of Ni25 coating by adding a mixture of ZrO_2_ and C to the Ni25 powder, and the ceramic composite coatings were reinforced by in situ laser synthesis on AISI 1045 steel substrate. The microstructure, phase formation, microhardness and wear resistance of the composite coating were examined. Experimental results show that the composite coating consists of γ-Ni, massive ceramic particles ZrC, NiZr_2_, Ni_7_Zr_2_, (Fe, Ni)_23_C_6_ and Fe_3_C. The in situ synthesized ZrC particles were evenly distributed in the composite coating. It was found that fine in situ ZrC particles improve the microhardness of the composite coating to 650 HV0.2, which is almost 2.7 times compared to the Ni25 coating.

In paper [46], the authors presented the results of selective laser remelting with a mixture of ZrB_2_, ZrC and B_4_C powders on a metallic tungsten substrate. A laser power of 200 to 400 W was used to apply the ceramic coatings. The resulting coatings were found to have mismatched thermal expansion with the substrate, which was detrimental to the integrality of the coating. The analysis of the microstructure and composition of the obtained coatings was used to explain the effect of thermal gradients and the influence of tungsten impurities on the solidification of ceramic coatings. In paper [47], the authors using a fiber laser obtained thin layers of 100% dense zirconium carbide ZrC on the surface of porous ZrC, the porosity of which was 30%. It was demonstrated experimentally and theoretically that oxidation to zirconium oxide is completely avoided at operating temperatures (at least 3420 °C). This type of highly refractory material—dense on the surface and porous in mass—can be used in high temperature applications where both diffusion properties as well as a thermal barrier are needed. However, these were only preliminary studies, and the authors did not develop this thread in further publications.

There are very few scientific publications in which the authors focus on the use of ZrC zirconium carbide. Due to the fact that cermets containing ZrC and TaC are characterized by very high hardness, it is reasonable to check whether these two carbides can be used as reinforcing phases in coatings produced on steel. The author made an initial attempt in a previous study by producing single laser tracks reinforced with TaC tantalum carbide [12]. Preliminary tests showed a possibility of producing Fe/TaC coatings on a steel substrate, and the obtained coating hardness values exceeded the hardness of the substrate. In this study, the author made an attempt to produce coatings with the second component of the aforementioned cermetal, i.e., ZrC zirconium carbide. The paper presents preliminary studies on the surface conditions and microstructure (Section 3.1) conducted using stereoscopic microscope and scanning electron microscope, chemical composition (Section 3.2) using EDS mapping and linear analysis as well as microhardness (Section 3.3). Basic studies were therefore carried out on single laser tracks, which may be new in the field of metal surface engineering.

## 2. Materials and Methods

In this study specimens made of 145Cr6 steel in the form of tiles with dimensions of 20 × 20 × 8 mm were used as the substrate. The chemical composition of steel used is presented in Table 1 and is in accordance with certificate delivered by the manufacturer.

**Figure 1:**
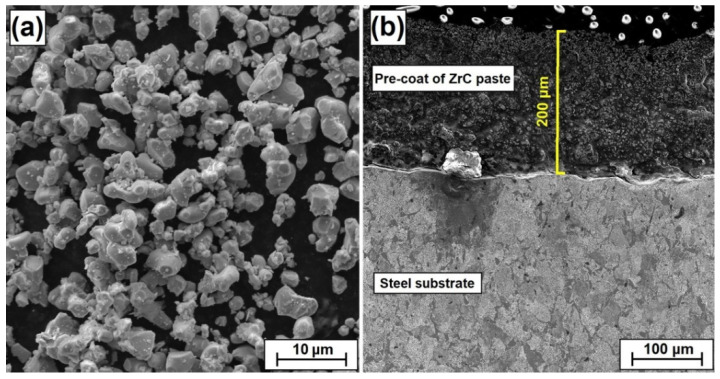
Morphology of zirconium carbide powder with average particle size less than 15 μm (**a**), zirconium carbide pre-coat applied on steel (**b**).

The shape and size of zirconium carbide powder particles was observed using scanning electron microscopy (SEM) and is presented in Figure 1a. The average particle size (APS) was less than 15 µm, which was in accordance with the delivered certificate. The powder purity was 99.9%. All presented parameters were in accordance with the producer data (Sigma-Aldrich, Saint Louis, MO, USA). In the first step, ZrC paste was produced. After applying this paste to a steel substrate, it formed a pre-coat. This paste consisted of ZrC powder, adhesive material in the form of sodium water glass as well as distilled water. In paste production, special attention was paid to its consistency, which has an influence on the possibility of its application to a steel substrate. Paste was prepared using 10 g (62.50 wt.%) zirconium carbide, 3 g (18.75 wt.%) water glass and 3 g (18.75 wt.%) distilled water. The second step was to apply the prepared paste to the steel substrate. For this purpose, the brush painting technique was used. The thicknesses of all pre-coats applied were measured. Due to an imperfect method of pre-coat application, some specimens had to be rejected. The specimens with a pre-coat thickness of 200 µm were intended for the study. The thickness of the coatings was measured by a PosiTector^®^ 6000 Advance ultrasonic thickness sensor (DeFelsko, New York, NY, USA) with an accuracy of ± 2 µm. Additionally, microscopic observations were made and exemplary thicknesses of pre-coats were measured (Figure 1b).

**Figure 2:**
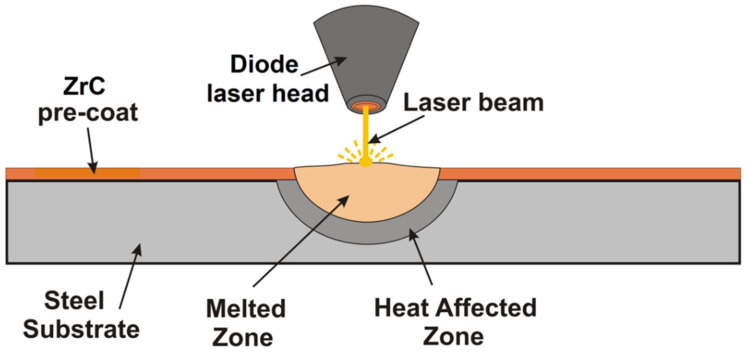
Schematic of Fe/ZrC coating production using laser processing.

The applied pre-coats were dried at ambient temperature for 24 h. After drying, specimen surfaces were subjected to laser processing. Laser processing was performed using a diode laser device—TruDiode 3006 (TRUMPF, Ditzingen, Germany) with a rated power of 3 kW. During the surface modification process, four different laser beam powers—500 W, 700 W, 900 W and 1100 W—were applied. The scanning speed of the laser beam was constant for all specimens and was 3 m/min, while the laser beam diameter was 1 mm. The distance between the tip of the laser head and the surface of the specimen (substrate) was constant and was equal to 60 mm. The schematic of Fe/ZrC coating formation with a single laser track is shown in Figure 2. Observations of the produced Fe/ZrC coatings were made both with the naked eye, using a magnifying glass as well as with the use of a stereoscopic microscope Leica EZ4 (Leica, Wetzlar, Germany). The surface roughness profiles reconstruction were determined on SEM images with Mountains^®^ SEM software manufactured by Digital Surf company (Digital Surf Headquarters, Besançon, France). Microstructure observations were carried out using a MIRA-3 scanning electron microscope (TESCAN, Brno, Czech Republic) on etched cross sections perpendicular to the produced Fe/ZrC coatings. To prepare specimens for observation, they were all ground and polished using a Mecatech 250 device (PRESI, Eybens, France) using grinding and polishing discs according to the manufacturer’s recommendations. Finally, specimens were etched in 5% HNO_3_ solution for 45 s to make the microstructure better visible. Chemical composition analyses were performed using an Ultim Max 65 energy dispersive spectrometer (Oxford Instruments, High Wycombe, UK) and Aztec Energy Live Standard software. Microhardness of produced Fe/ZrC coatings was studied with FM-810 microhardness tester (Future-Tech, Kawasaki, Japan). Indentation dimensions were measured using FT-Zero automatic indentation measuring software from the same company. Microhardness tests were carried out using an indentation load of 50 g, while the loading time was 15 s. Measurements were made from the surface to the substrate in a matrix in which no unremelted ZrC particles were found (only secondary carbide dendrites were present there). In addition, measurements were also made in areas where there were many fine particles of unremelted ZrC in the form of agglomerates.

## 3. Results and Discussion

### 3.1. Surface Condition and Microstructure

**Figure 3:**
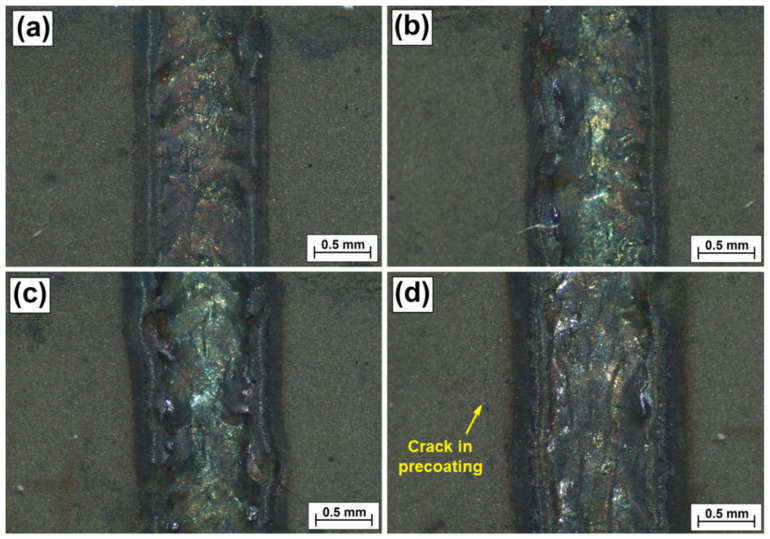
Surface condition of Fe/ZrC coatings—single laser tracks produced using laser beam power of 500 W (**a**), 700 W (**b**), 900 W (**c**) and 1100 W (**d**).

Figure 3 shows the surface condition of the produced Fe/ZrC coatings in the form of single laser tracks directly following laser processing. It was found that laser beam power has an influence on their width, which becomes larger with an increase in beam power. The applied paste-form ZrC precoating can be seen on the sides of laser tracks generated. As can be observed, this coating has an even surface, without sticking, furrows or gas bubbles. There are also no traces of the brush painting process. Cracks were observed in some areas of the precoating (e.g., in Figure 3d—along the laser track on the left). However, it was found that such a defect does not have a considerable impact on laser beam processing. No cracks were observed in the produced laser tracks; however, their surface was not uniform. The photos in Figure 3 were taken immediately after the Fe/ZrC coatings were created with a laser beam. The ZrC precoating was chipped when samples were cut to make cross-sections for microstructure evaluation. This is the reason why there is no precoating in the microstructure photos shown later in this article.

**Figure 4:**
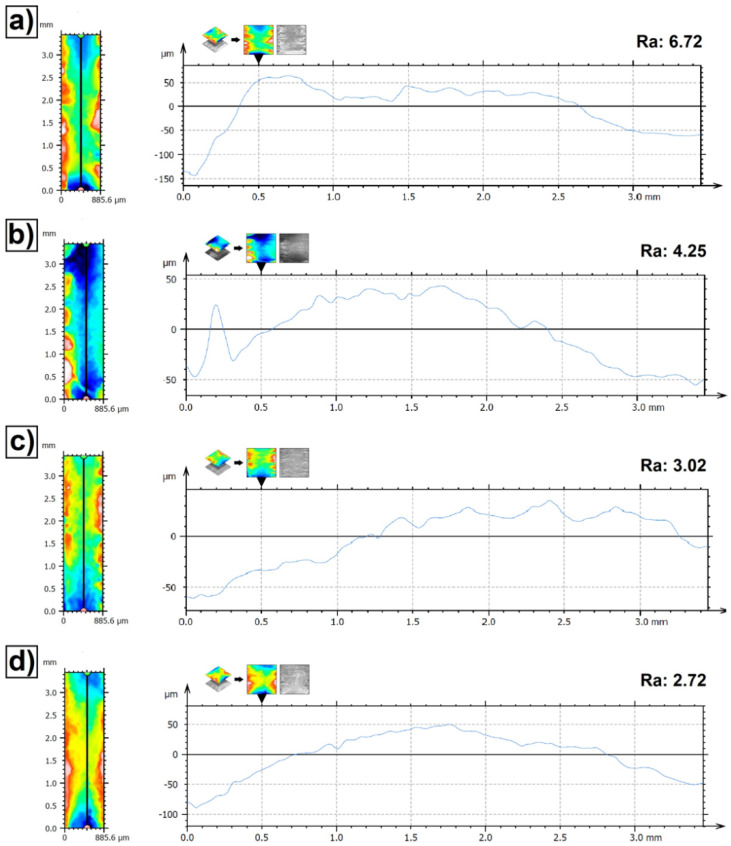
Surface roughness of Fe/ZrC coatings—single laser tracks produced using laser beam power of 500 W (**a**), 700 W (**b**), 900 W (**c**) and 1100 W (**d**).

Figure 4 shows the surface roughness of the produced Fe/ZrC coatings. All measurements were made in the axis of the laser tracks. The parameters Ra (arithmetic mean profile deviation) was analyzed. The surface roughness Ra gradually decreases as the power of the laser beam increases. By increasing the power of the laser beam from 500 W to 1100 W, the roughness was reduced about 2.5 times. This was due to the application of more heat to the precoating which solidified a little longer as final coating. At the same time, the ZrC particles melted to a greater extent with the Fe matrix.

**Figure 5:**
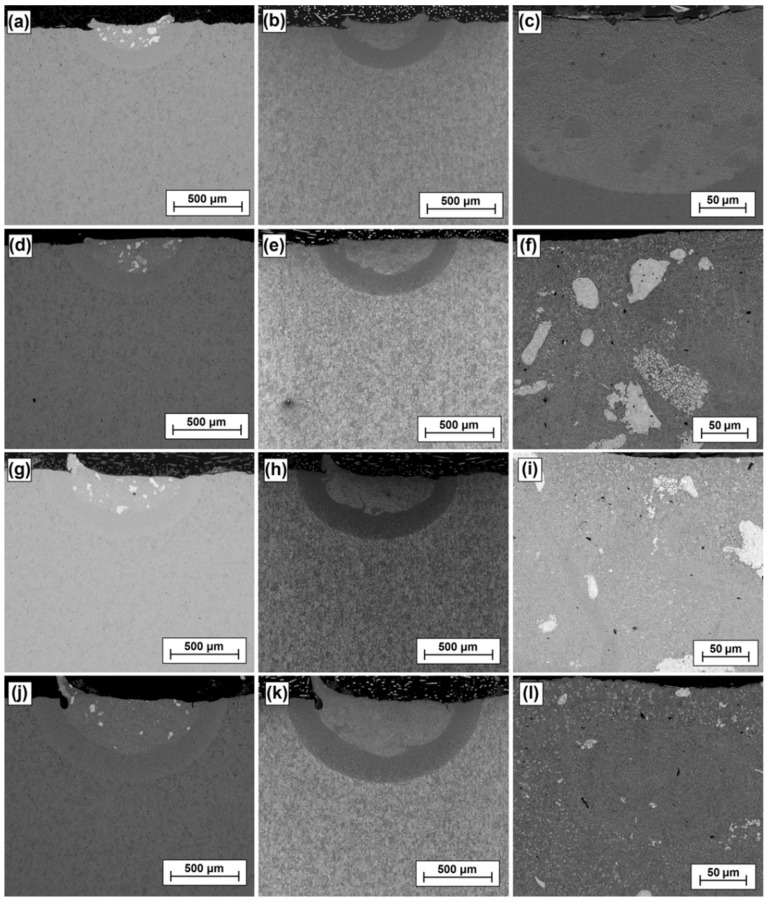
Morphology of Fe/ZrC coatings produced using laser beam powers of 500 W—BSE image (**a**); 500 W—SE image (**b**); 500 W—magnification of melted zone (**c**); 700 W—BSE image (**d**); 700 W—SE image (**e**); 700 W—magnification of melted zone (**f**); 900 W—BSE image (**g**); 900 W—SE image (**h**); 900 W—magnification of melted zone (**i**); 1100 W—BSE image (**j**); 1100 W—SE image (**k**); 1100 W—magnification of melted zone (**l**).

Figure 5 shows a view of the ZrC composite coatings produced with four laser beam powers. As a result of diode laser beam action on sample cross-sections, three characteristic zones were obtained. A remelted zone was created at the surface and a heat-affected zone just below it. Both these zones were characterized by a parabolic zone boundary line formed by action of the heat. The remelted zone was created as a result of remelting a steel substrate with a ZrC initial coating applied in paste. The third zone is the core of the material with an unchanged microstructure (steel substrate). In Figure 4, the SEM images are presented in three columns. The left column (Figure 5a,d,g,j) shows the morphology of the coatings made in backscattered electron (BSE) contrast. The middle column (Figure 5b,e,h,k) shows morphology of the coatings in secondary electrons contrast (SE). The right column is a magnification of the remelted zone. It was found that dimensions of tracks produced increased with increasing laser beam power. Both the thickness of the remelted zone and the heat affected zone increased.

With increasing power, the remelted zone increased in thickness from about 190 µm at 500 W, to about 250 µm (700 W) and 280 µm (900 W) up to about 410 µm for a coating produced at 1100 W. The thickness of the heat-affected zone increased much less as the values were: 150 µm, 160 µm, 185 µm and 204 µm respectively. In the upper part of some coatings, characteristic burrs are visible which were formed by fusing the pre-coating and the lack of remelting of this coating with the substrate on the sides of the track. This problem should be eliminated by making multiple tracks. As seen in Figure 5, the part of the precoating that was not remelted fell off in sample preparation (during cutting). This was due to a rather poor adhesion of the precoating to the steel substrate. However, the precoating adhesion is good enough and it is expected that when making multiple tracks it will continue to function as a surface modifier and will not fall off in laser processing, which had been repeatedly observed by the author in earlier studies. A composite structure was observed in all the analyzed coatings as agglomerates of ZrC particles in an iron-based matrix were obtained. They are visible as white spots against a dark background. These agglomerates were characterized by a very diverse structure and size. Some of them were compact with a structure similar to the sintered material. Elsewhere, the agglomerates were less compact and resembled a coating produced by laser cladding with powder. The idea behind producing Fe/ZrC coatings was to obtain coatings with a most homogeneous structure and of a composite nature. The high-melting ZrC powder particles (3540 °C) were meant to strengthen the iron-based matrix characterized by a lower melting point (1538 °C). It is the ZrC phases that give the coating its high hardness. With increases in laser beam power, the number of ZrC agglomerates decreased. A significant advantage of the produced Fe/ZrC coatings is their very good bonding with the steel substrate, which can be described as metallurgical. Such good bonding occurs along the entire length of the parabolic remelting line. No porosity or cracking of the coating were found, despite the fact that a very hard material was used as the reinforcement phase. The obtained results induce the author to create multiple tracks and subject them to a detailed analysis.

**Figure 6:**
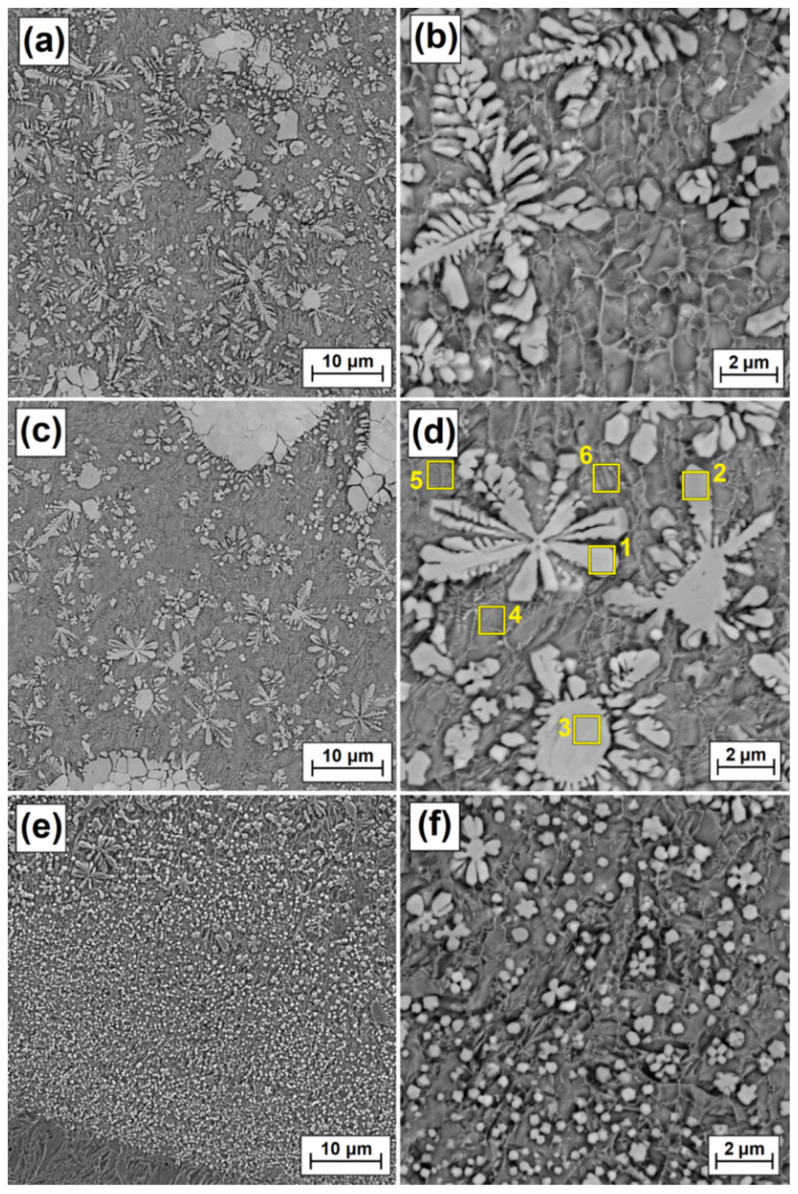
Melted zone microstructure of Fe/ZrC coatings produced using a laser beam power of 500 W; subsurface area (**a**), magnification of subsurface area (**b**), middle area (**c**), magnification of middle area (**d**), sub-substrate area (**e**), magnification sub-substrate area (**f**).

**Figure 7:**
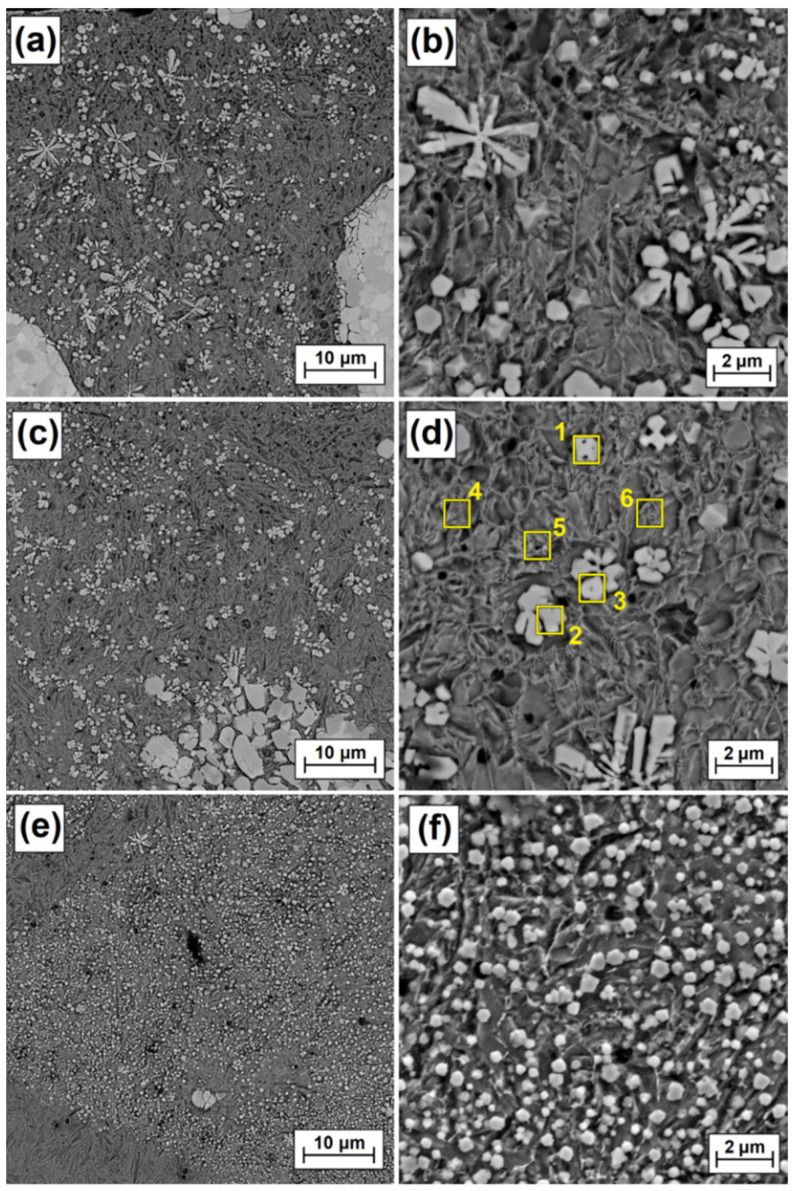
Melted zone microstructure of Fe/ZrC coatings produced using laser beam power of 700 W; subsurface area (**a**), magnification of subsurface area (**b**), middle area (**c**), magnification of middle area (**d**), sub-substrate area (**e**), magnification sub-substrate area (**f**).

**Figure 8:**
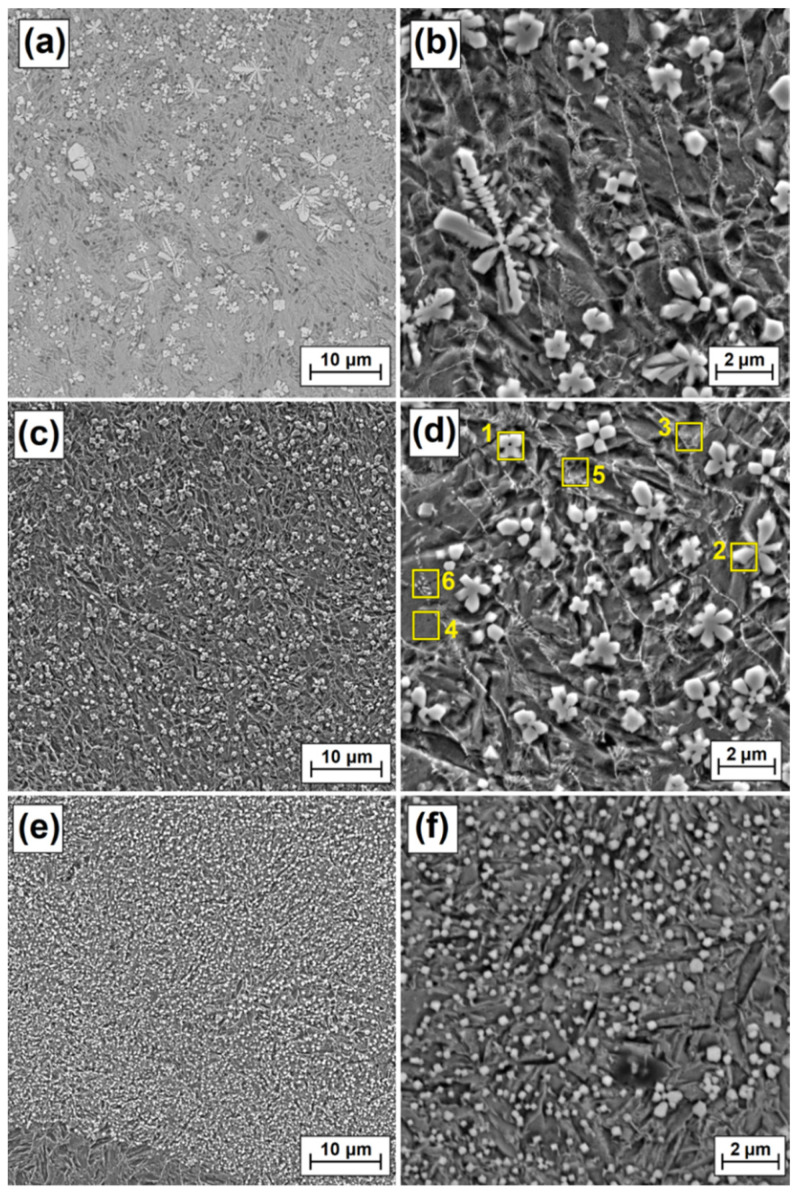
Melted zone microstructure of Fe/ZrC coatings produced using laser beam power of 900 W; subsurface area (**a**), magnification of subsurface area (**b**), middle area (**c**), magnification of middle area (**d**), sub-substrate area (**e**), magnification sub-substrate area (**f**).

**Figure 9:**
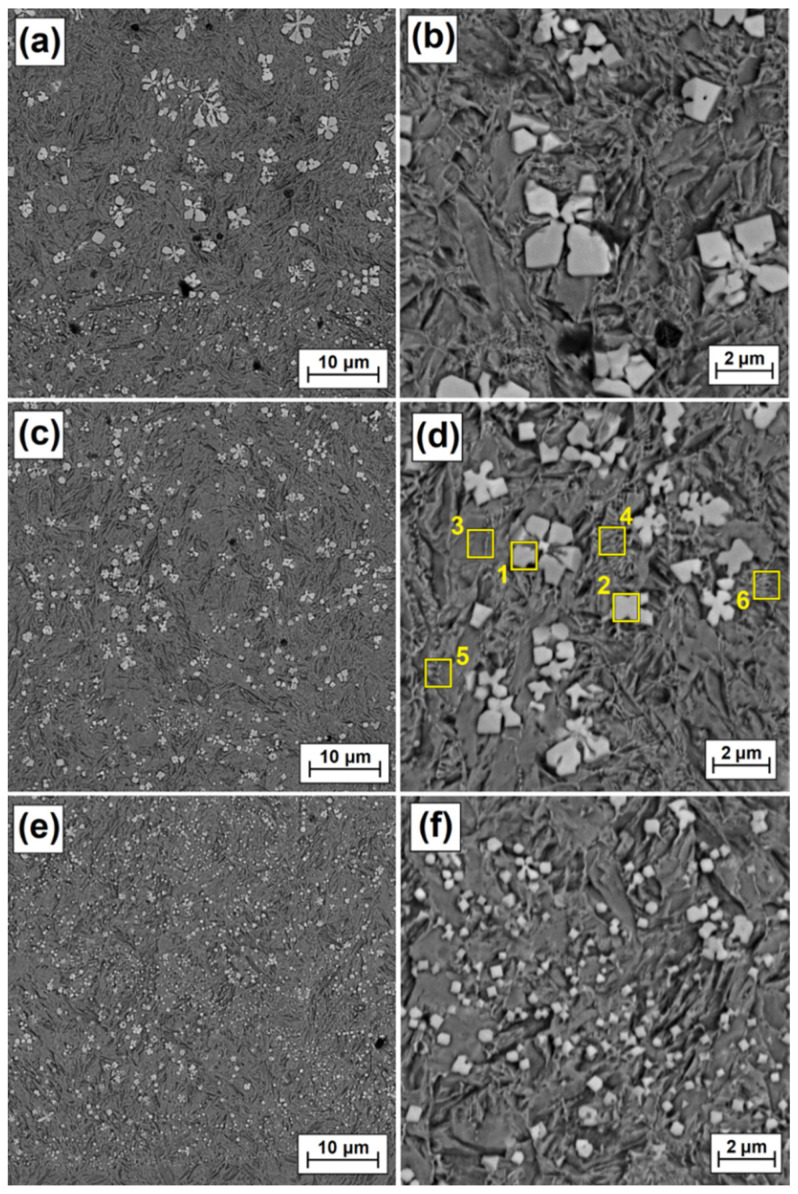
Melted zone microstructure of Fe/ZrC coatings produced using laser beam power of 1100 W; subsurface area (**a**), magnification of subsurface area (**b**), middle area (**c**), magnification of middle area (**d**), sub-substrate area (**e**), magnification sub-substrate area (**f**).

Figure 6, Figure 7, Figure 8 and Figure 9 show the microstructure of Fe/ZrC coatings produced by laser processing of a paste precoating of 200 µm. The figures show successively the areas of the remelted zone: top (near-surface), middle and bottom (sub-substrate), and next to them magnifications of selected fragments of the microstructure. The test results are presented for the coatings produced with the use of a 500 W laser beam (Figure 6), 700 W (Figure 7), 900 W (Figure 8) and 1100 W (Figure 9). As for the Fe/ZrC coating, the characteristic dendritic carbide precipitations were observed at a laser beam power of 500 W (Figure 6a) They were formed as a result of the complete remelting of fine ZrC particles and their recrystallization in an iron-based matrix. The presence of unmelted zirconium carbide agglomerates was also found. Dendritic separations were the largest at the surface of the remelted zone. They were characterized by long primary dendrite arms and the presence of secondary dendrite arms (Figure 6b) As the distance from the surface increases (middle zone, Figure 6c) the size of the dendritic carbide precipitates decreased and the second-order arms disappeared (Figure 6d). This was due to the reduction of laser beam heat. The heat delivered to the lower zones of the Fe/ZrC coating produced at a beam power of 500 W (Figure 6e) was very quickly removed by the steel substrate, so that no large amount of dendrite precipitates was observed there. The growth of dendritic phases was inhibited by a sudden heat removal. The lower part of the coating is characterized by a typical iron-based composite microstructure and flower-shaped separations (Figure 6f). In the coating under discussion, no cracks or porosity were found even when viewed at very high magnification.

The microstructure of the remelted zone, obtained with a laser beam power of 700 W, looks very similar (Figure 7a). However, an increased laser beam power resulted in a greater remelting of the substrate, and thus a greater share of iron in the produced Fe/ZrC coating. This affected the number, size and shape of the dendritic carbide precipitates. The primary dendrite arms are much shorter, and no second order dendrites are observed (Figure 7b). This is probably caused by very fast solidification of the matrix and thus blockage of carbide growth. The matrix, which was more iron-enriched than the coating made at 500 W, tended to solidify much more quickly. The greater amount of iron lowered the total remelting point of coating matrix. In Figure 6a agglomerates of ZrC particles and their structure are clearly visible. The etching revealed a granular structure of these agglomerates. It is pure zirconium carbide that was not remelted, but most likely was sintered in high temperatures. As it moved away from the coating surface, as previously, the cooling rate of the remelted zone increased, which resulted in an even greater reduction in both the amount of dendritic precipitates (Figure 7c) and their dimensions (Figure 7d). Finally, at the very steel substrate, a composite microstructure was obtained (Figure 7e), which consisted of an iron-based matrix and reinforcing phases in the form of polygon-shaped secondary carbide precipitates (Figure 7f). There are fine porosities in the coating, but their amount is very small. A very good bonding was observed between a lower area of the remelted zone and the martensitic heat-affected zone (lower area in Figure 7e).

As laser beam power increased, the reduction in carbide precipitate dimensions was observed more and more clearly. The use of laser beam power of 900 W contributed to a significant reduction in the amount of dendritic carbide precipitates in the subsurface zone (Figure 8a). Separations in the form of polygons (Figure 8b) or stars are prevalent there. In the area located in the central part of the remelted zone (Figure 8c), these separations are small, not exceeding 2 µm (Figure 8d). Local occurrence of eutectics was observed between the separations. In the zone farthest from the coating surface (Figure 8e), i.e., where the substrate acts as a cooler, secondary carbide precipitates are the smallest, with their dimensions often not even reaching 0.5 µm. This area can be characterized as a fine dispersion composite system.

In the last analyzed coating, i.e., the one obtained by processing ZrC precoating with 1100 W laser beam power, no typical dendritic carbide precipitates were observed (Figure 9a). Rather, they are polygons with a shared crystallization nucleus (Figure 9b,d). Due to the highest (among those used) power of the laser beam, a very high remelting was found, and thus an increased iron share in the produced Fe/ZrC coating. Thus, the share of secondary carbide phases in all zones, both in the middle zone, decreased significantly (Figure 9c) as well as in the zone near the steel substrate (Figure 9e). It was found that the matrix is most likely made of martensite needles, as coating microstructure is similar to that of the heat-affected zone. The cooling rate of the remelted zone closest to the steel substrate contributed to the formation of separations often much smaller than 0.5 µm.

### 3.2. Chemical Composition

Table 2 presents the results of chemical composition measurements using the EDS method for individual measurement areas marked in Figure 6d (Fe/ZrC coating produced using 500 W), Figure 7d (Fe/ZrC coating produced using 700 W), Figure 8d (Fe/ZrC coating produced using 900 W) and Figure 9d (Fe/ZrC coating produced using 1100 W). Characteristic and repeatable areas were selected for the tests, both for the iron-based matrix and for carbide precipitates. The obtained data confirmed that the dendritic carbide phases were released in the coating. The dendritic arms were therefore complex carbide phases composed of components derived from the substrate as well as from zirconium carbide, which is the coating modifier. It can also be seen from Table 2 that the matrix consists mainly of iron, but certain small amounts of zirconium or zirconium carbides are observed in it. The exact phase composition should be confirmed using the XRD method, but due to the production of only single laser tracks in the preliminary studies, this was not possible. Studies in this area will be continued on multiple tracks and will be presented in the author’s future papers.

**Figure 10:**
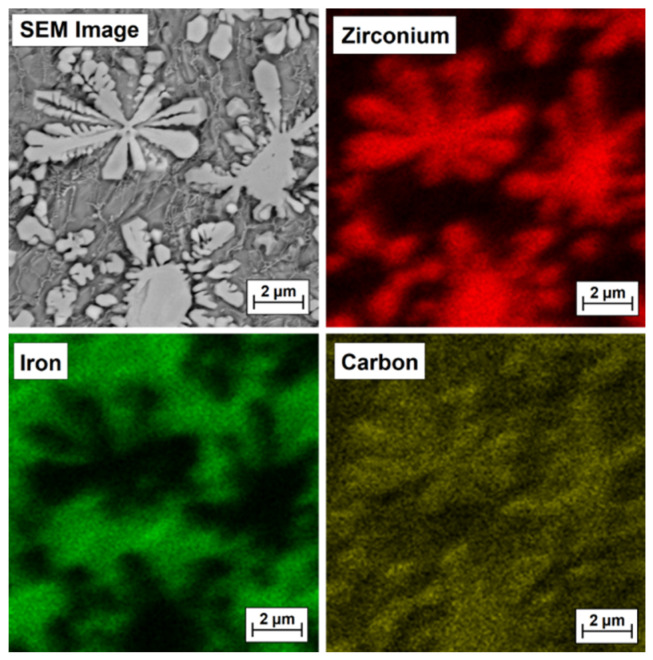
Microstructure and EDS mapping of Fe/ZrC coating produced using a laser beam power of 500 W—middle area of melted zone.

**Figure 11:**
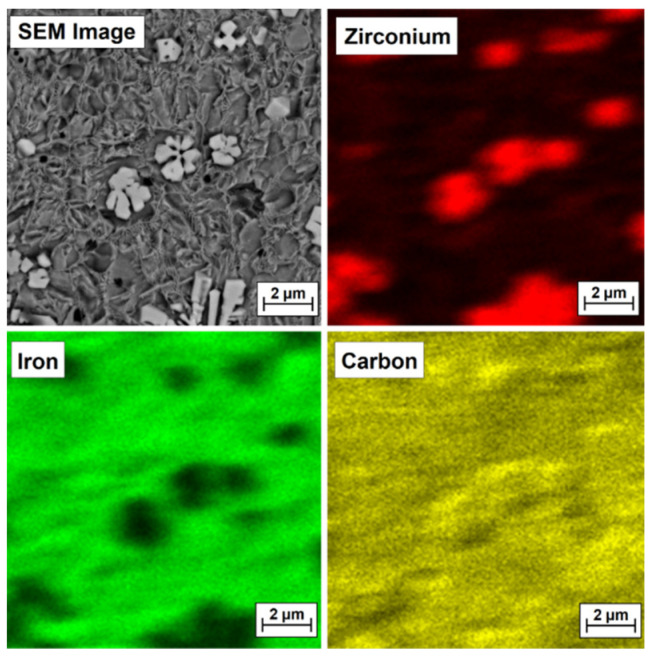
Microstructure and EDS mapping of Fe/ZrC coating produced using a laser beam power of 700 W—middle area of melted zone.

**Figure 12:**
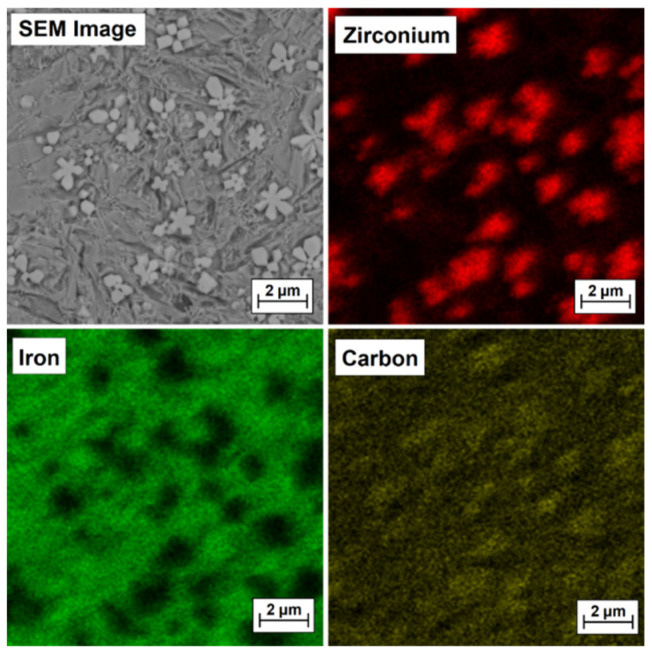
Microstructure and EDS mapping of Fe/ZrC coating produced using a laser beam power of 900 W—middle area of melted zone.

**Figure 13:**
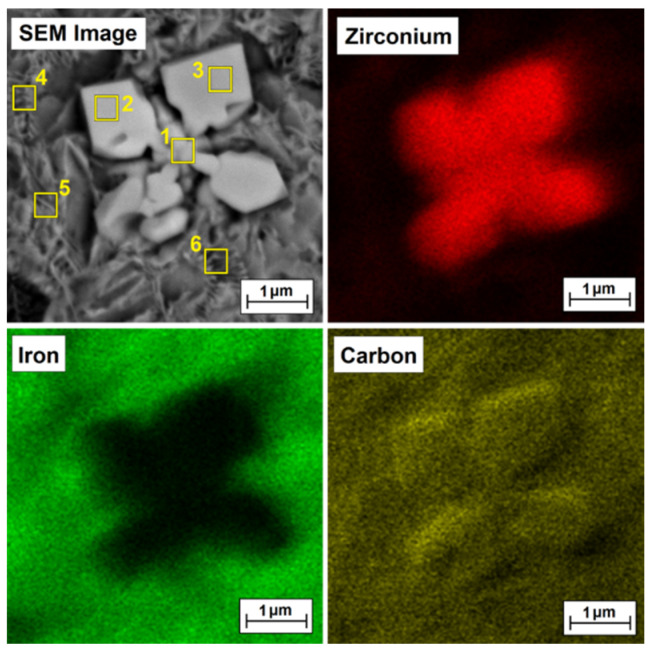
Microstructure and EDS mapping of Fe/ZrC coating produced using a laser beam power of 1100 W—middle area of melted zone.

To confirm that bright precipitates in the microstructure of the coatings are secondary or primary zirconium carbides or zirconium-containing complex carbides, EDS mapping was performed, and the test results are presented in Figure 9, Figure 10, Figure 11 and Figure 12 for the Fe/ZrC coating produced at 500 W (Figure 10), 700 W (Figure 11), 900 W (Figure 12) and 1100 W (Figure 13). The sites where zirconium was identified are marked in red. As for Fe/ZrC coating produced with the use of 1100 W laser beam power, an EDS map was made in the area of a flower-shaped secondary carbide precipitation. In addition to making a standard map, which clearly shows that this separation is mainly composed of carbon and zirconium, the chemical composition was also tested in the areas marked in Figure 13.

The test results are shown in Table 3. It can be concluded that the richest in zirconium is the middle part of the separation, i.e., its nucleus, in which 78.5 wt.% Zr was found. Slightly less zirconium is found in the “flakes” of the separation. In the matrix area, a predominant iron share was found. However, it should be remembered that the coatings were not produced on pure iron, but on steel containing other elements that were not analyzed, and the EDS method itself divides the analyzed elements in such a way that the sum of their share amounts to 100%. Nevertheless, this study proved that it is possible to produce composite coatings where an iron alloy is a matrix for a zirconium carbide phase.

**Figure 14:**
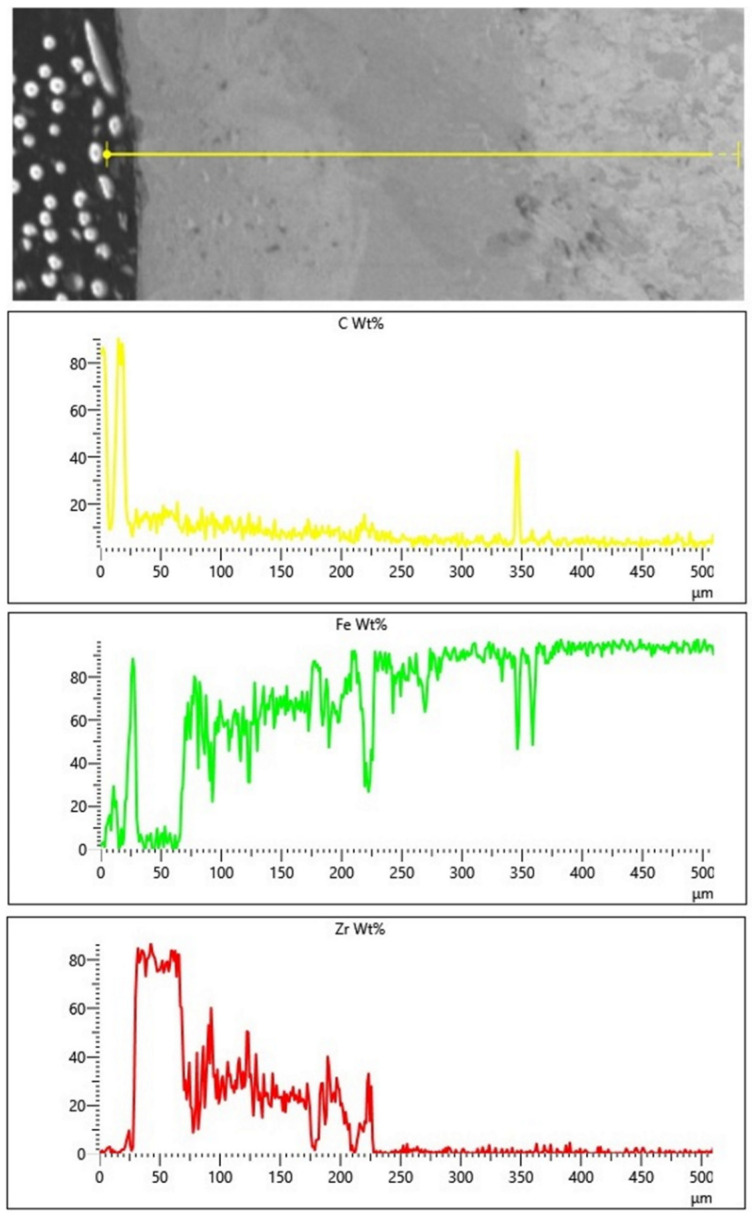
Linear EDS analysis on cross section of Fe/ZrC coating produced using laser beam power 500 W.

**Figure 15:**
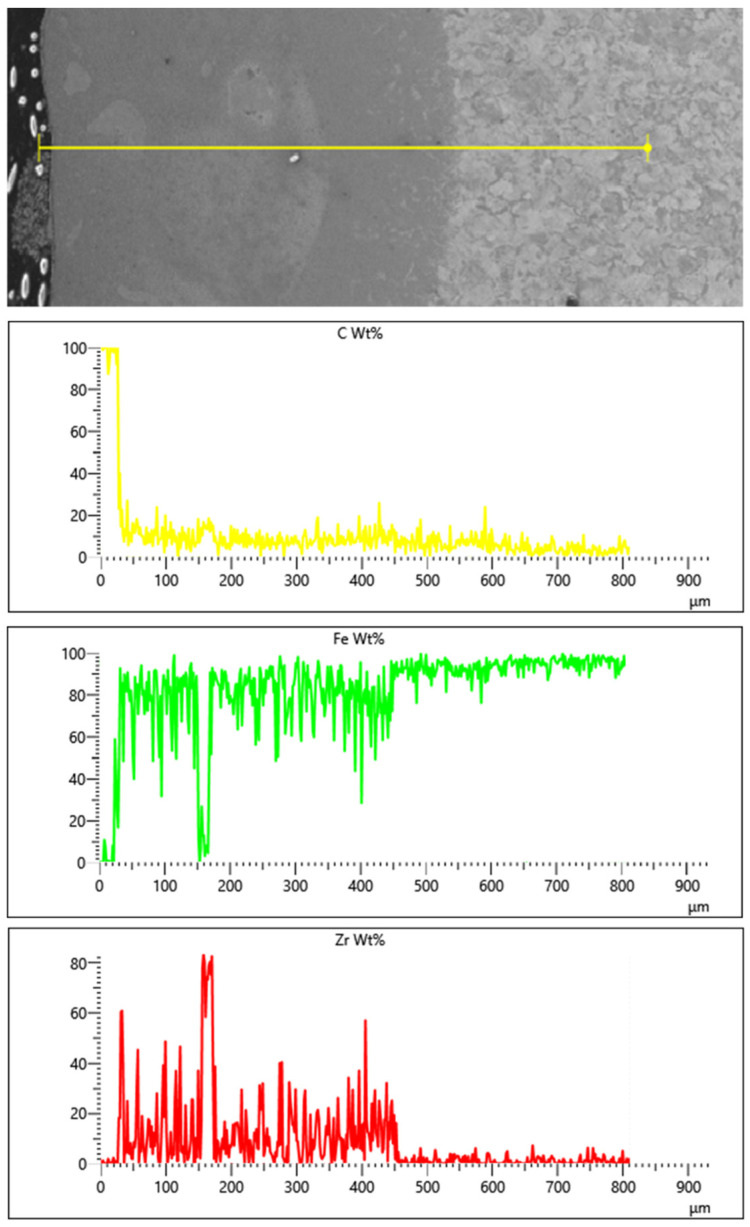
Linear EDS analysis on cross section of Fe/ZrC coating produced using laser beam power 1100 W.

Additionally, a linear EDS analysis for Fe/ZrC coatings was performed. The limitation of this method is analyzing only the area along a given line. For composite coatings, the results of this test depend on covering the individual components of the composite (matrix and reinforcing phase) with the test line. This paper includes only exemplary test results for the extreme values of the laser beam power: 500 W (Figure 14) and 1100 W (Figure 15). Due to increased carbon content in the areas of occurrence, zirconium carbides in the form of agglomerates was found. The presence of zircon throughout the entire thickness of the remelted zone was determined. This is confirmed by both the EDS mapping and the microstructure tests. It is planned to conduct a detailed analysis of EDS, EBSD and XRD on coatings made with the same parameters but in the form of multiple tracks.

### 3.3. Microhardness

**Figure 16:**
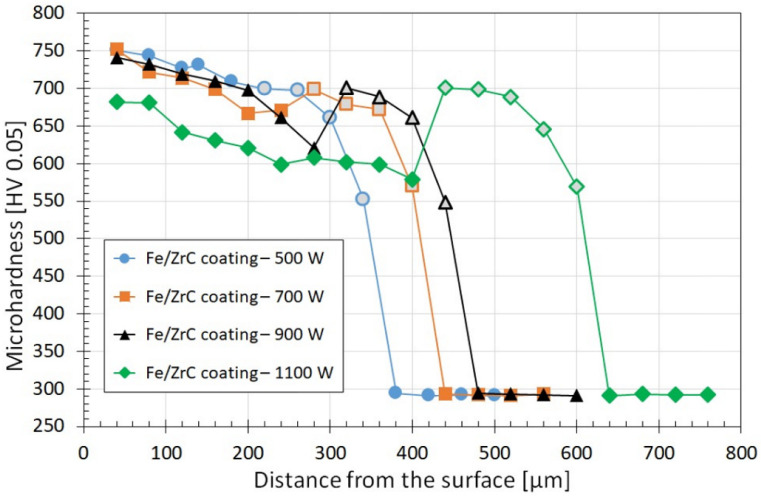
Microhardness of Fe/ZrC coating produced on steel using different laser beam power-markers with a gray background on the graph indicating that the measurements are in the heat-affected zone.

Figure 16 shows the results of microhardness tests of Fe/ZrC coatings produced with varying diode laser beam powers. The studies necessary to prepare the graphs were carried out only on the iron-based matrix, omitting large precipitates of carbide phases. The impact of laser beam power on the microhardness of the remelted zone and a negligible influence of laser beam power on the microhardness of the heat-affected zone was found. Microhardness of the remelted zone decreases with increasing laser beam power, which is a result of increasing iron share in this zone. The matrix hardness is in most cases similar to the hardness of the heat affected zone and is approximately 750 HV0.05 (Fe/ZrC, 500 W) up to approximately 635 HV0.05 (Fe/ZrC, 900 W). An exception is the Fe/ZrC coating produced with a laser beam of 1100 W. It was found there that the heat-affected zone is harder than the matrix of the remelted zone, regardless of the distance from the sample surface. The minimum microhardness of the matrix in the remelted zone is about 580 HV0.05, with the hardness of the heat affected zone at the level of 700 HV0.05. In the coatings produced with laser beam powers of 700 W and 900 W, a sudden increase in the microhardness at the transition to the heat-affected zone was also found; however, in these cases, in the iron-based matrix, hardness values higher than in this zone were detected at the surface. However, it should considered that microhardness measurements shown in Figure 16 concern solely the sites where the matrix occurs, without a significant share of carbide precipitates and without measurements of agglomerate hardness. Therefore, measurements of microhardness were performed in sites with an increased number of dendritic carbide precipitates and microhardness of agglomerates. Twenty measurements were made for each coating at each of these locations, and then they were averaged. In the sites where a very large amount of dendritic carbide precipitates occurs, an average hardness of approximately 890 HV0.05 was obtained, while average agglomerate hardness is approximately 1980 HV0.05. It can therefore be concluded that the average hardness of the produced coatings is much higher than that shown in Figure 16.

**Figure 17:**
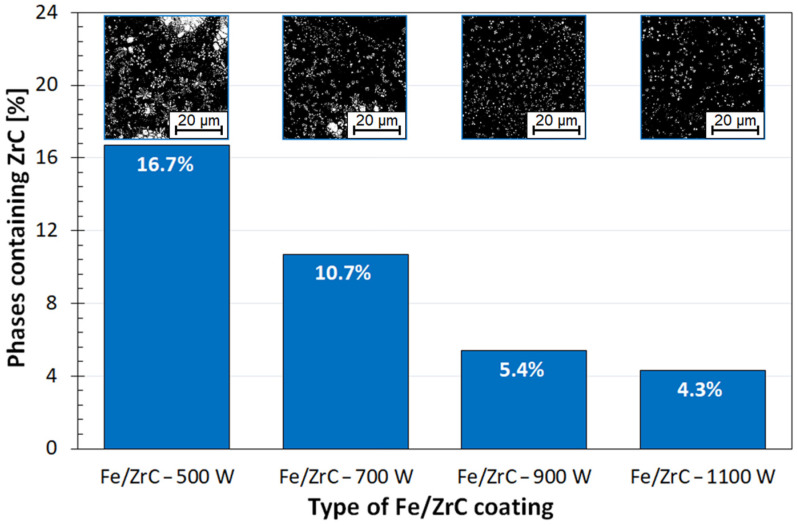
Graph of the content of the carbide phase in the Fe-based matrix—the figures above the bars show the carbide phase (white color) and the matrix (black color) for the area in the center of the remelted zone.

However, this hardness is not evenly distributed, which makes it difficult to clearly present test results in a graph, as the graph would be very irregular and darkened. Therefore, it was decided that beside presenting the microhardness profile, a graph showing a percentage of hard carbide phases in individual coatings will also be presented (Figure 17). This diagram shows how much of the middle part of the remelted zone is carbide phases. The graph also includes photos of these zones, converted to black and white images. As a result, the number of carbide phases (white areas) and matrix (black areas) were determined in a fairly precise manner. According to the author, such a graph as presented in Figure 17 better illustrates actual coating microhardness, which may affect other properties, e.g., wear resistance.

**Figure 18:**
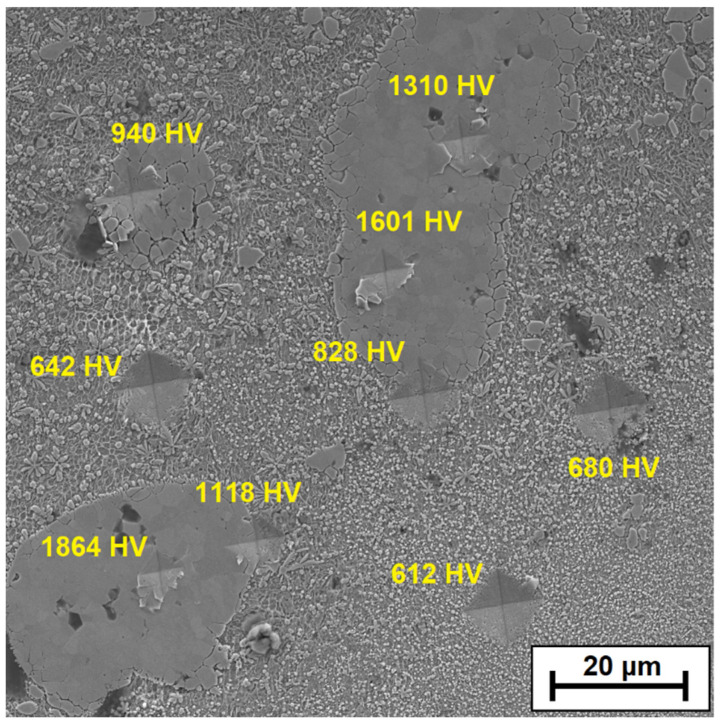
Examples of microhardness indentations for the reinforcing phase and the matrix—the middle part of remelted zone of Fe/ZrC coating produced using a laser beam power of 700 W.

Additionally, Figure 18 shows the selected area of the remelted zone for the coating produced with the laser beam power of 700 W. In this area, microhardness indentations were made using the Vickers method at a load of 50 g. Measurements were made and a significant difference in hardness between the ZrC agglomerates and the matrix containing carbide precipitates was found. The measurement results were marked on the SEM image.

## 4. Conclusions

The main findings of the studies conducted are as follows:

1. It is possible to produce a composite Fe/ZrC coating, where the matrix is an iron-based alloy (steel) and the carbide phase is the reinforcing phase. In addition, it is possible to produce such a coating by remelting the precoating on steel with a paste in which zirconium carbide is the main component. The study was carried out on single laser tracks, which is a kind of limitation. However, the obtained results bode well for the production of full-value coatings in the form of multiple laser tracks on entire area of substrate.
2. The laser beam power used is very important in the production of Fe/ZrC coatings, as it determines production of the composite microstructure and allows a smaller or larger number of carbide phases to be obtained. The amount of reinforcing phase can have a significant influence on the final properties of the coatings.
3. Remelting finer particles of ZrC powder leads to precipitation of secondary carbides in the iron-based matrix, which increases coating microhardness.
4. The greatest hardness can be achieved using the highest possible proportion of non-remelted or only partially remelted zirconium carbides.
5. As laser beam power increases, the proportion of carbide phase decreases, which results in a reduction in the hardness of Fe/ZrC coating produced. One limitation is the ability to unequivocally determine the microhardness of the coating, because there are large differences in the microhardness of the matrix and the ZrC particles present in it.

## Figures and Tables

**Table 1:** Chemical composition of steel used in this study [wt.%].

C	Mn	Si	P	S	Cr	V	Fe
1.35	0.60	0.30	0.02	0.02	1.45	0.20	bal.

**Table 2:** Chemical composition (EDS) for Fe/ZrC coating produced using different laser beam power [wt.%].

Type of Coating	No	Zr	Fe	C
Fe/ZrC 500 W	1	70.3	15.0	14.7
	2	46.5	38.5	14.9
	3	80.2	2.9	16.9
	4	9.0	84.2	6.8
	5	8.0	84.1	7.9
	6	10.0	83.4	6.7
Fe/ZrC 700 W	1	66.9	14.6	18.4
	2	64.6	17.9	17.5
	3	53.0	27.4	19.6
	4	12.6	69.2	18.2
	5	12.5	71.7	15.8
	6	6.2	82.1	11.7
Fe/ZrC 900 W	1	59.0	24.3	16.7
	2	34.6	50.6	14.9
	3	6.0	86.5	7.5
	4	2.0	90.7	7.4
	5	3.8	88.1	8.0
	6	4.3	89.2	6.5
Fe/ZrC 1100 W	1	51.5	33.6	14.9
	2	72.1	10.6	17.3
	3	1.2	92.5	6.3
	4	7.4	83.6	9.0
	5	11.0	78.8	10.2
	6	9.5	82.8	7.6

**Table 3:** Chemical composition (EDS) for selected area in Fe/ZrC coating produced using a laser beam power of 1100 W [wt.%].

Type of Coating	No	Zr	Fe	C
Fe/ZrC 1100 W	1	78.5	3.5	18.0
	2	68.9	11.4	19.7
	3	73.1	8.2	18.7
	4	10.1	74.9	15.0
	5	9.1	77.9.	13.0
	6	8.7	80.0	11.3

## Data Availability

Data available on request.
